# DRpred: A Novel Deep Learning-Based Predictor for Multi-Label mRNA Subcellular Localization Prediction by Incorporating Bayesian Inferred Prior Label Relationships

**DOI:** 10.3390/biom14091067

**Published:** 2024-08-26

**Authors:** Xiao Wang, Lixiang Yang, Rong Wang

**Affiliations:** 1 School of Computer Science and Technology, Zhengzhou University of Light Industry, Zhengzhou 450000, China; yanglixiang@email.zzuli.edu.cn; 2 Henan Provincial Key Laboratory of Data Intelligence for Food Safety, Zhengzhou University of Light Industry, Zhengzhou 450000, China; 3 School of Electronic Information, Zhengzhou University of Light Industry, Zhengzhou 450000, China; wangrong@zzuli.edu.cn

**Keywords:** mRNA, subcellular localization, multi-label prediction, Bayesian networks, BiLSTM

## Abstract

The subcellular localization of messenger RNA (mRNA) not only helps us to understand the localization regulation of gene expression but also helps to understand the relationship between RNA localization pattern and human disease mechanism, which has profound biological and medical significance. Several predictors have been proposed for predicting the subcellular localization of mRNA. However, there is still considerable room for improvement in their predictive performance, especially regarding multi-label prediction. This study proposes a novel multi-label predictor, DRpred, for mRNA subcellular localization prediction. This predictor first utilizes Bayesian networks to capture the dependencies among labels. Subsequently, it combines these dependencies with features extracted from mRNA sequences using Word2vec, forming the input for the predictor. Finally, it employs a neural network combining BiLSTM and an attention mechanism to capture the internal relationships of the input features for mRNA subcellular localization. The experimental validation on an independent test set demonstrated that DRpred obtained a competitive predictive performance in multi-label prediction and outperformed state-of-the-art predictors in predicting single subcellular localizations, obtaining accuracies of 82.14%, 93.02%, 80.37%, 94.00%, 90.58%, 84.53%, 82.01%, 79.71%, and 85.67% for the chromatin, cytoplasm, cytosol, exosome, membrane, nucleolus, nucleoplasm, nucleus, and ribosome, respectively. It is anticipated to offer profound insights for biological and medical research.

## 1. Introduction

The subcellular localization of RNA has become a very popular research topic in recent years, with the subcellular localization of messenger RNA (mRNA) playing a significant role in regulating gene expression [1,2,3,4,5,6]. The localization of mRNA within various subcellular regions allows for the targeting of the encoded protein and achieves precise quantitative and spatial control of the protein, including the regulation of ribosome availability and the specific locations of translation [7,8,9,10]. Furthermore, mRNA subcellular localization also plays a crucial role in many biological processes, including embryonic development, asymmetric cell division, cell polarity, cell migration, and neuronal morphogenesis [11,12,13,14]. This localization ensures precise temporal and spatial control over protein synthesis, thereby influencing cellular differentiation, tissue patterning, and the formation of complex multicellular organisms. It is noteworthy that researchers have also found that the occurrence of many diseases is associated with abnormalities in mRNA subcellular localization, such as neurodegenerative diseases, Fragile X syndrome, embryonic disorders, Alzheimer’s disease, and cancer [15,16,17,18,19]. Therefore, investigating mRNA subcellular localization not only assists in spatially regulating gene expression but also enhances our comprehension of how RNA localization patterns relate to the mechanisms underlying human diseases, offering significant implications for biology and medicine.

During the 1980s, researchers first observed the asymmetric distribution of specific mRNA in the cytoplasm when using in situ hybridization techniques to detect b-actin mRNA in ascidian embryos [20]. Subsequently, researchers further identified cases of differential localization in transcripts encoding cytoskeletal proteins in cultured chicken cells [21]. In recent years, techniques such as fluorescence in situ hybridization, APEX-RIP, and CeFra-seq have been employed to measure mRNA levels in specific subcellular locations, facilitating the analysis and assessment of its distribution patterns [22,23,24,25]. However, these biochemical experimental methods necessitate substantial time and financial resources, and the generation of extensive datasets during experiments presents challenges for analysis [26,27]. With the development of high-throughput RNA sequencing technologies, the quantity of mRNA transcripts is increasing exponentially, and this will drive and accelerate the development of low-cost, efficient new methods by researchers to address the current challenges faced in RNA localization [28,29,30].

In recent years, machine learning [31] and deep learning [32] have been widely applied to address various problems in the field of biology. In 2019, Yan et al. [33] developed the first mRNA localization predictor, RNATracker, based on deep neural networks. This predictor utilizes sequence and secondary structure information and integrates several state-of-the-art deep learning techniques, including CNNs, LSTMs, and attention layers, thereby advancing research in this field. In 2020, Garg et al. [34] developed mRNALoc, an SVM-based predictor that uses primary mRNA sequence information to predict five subcellular localizations of eukaryotic mRNA. In the subsequent year, a series of mRNA subcellular localization predictors emerged. For instance, Li et al. [35] developed SubLocEP, Tang et al. [36] developed mRNALocater, Musleh et al. [37] developed MSLP, and Zhang et al. [38] developed a subcellular localization predictor specifically targeting human mRNA. These predictors, utilizing machine learning and deep learning methods, can accurately predict the subcellular localization of mRNA, such as in the exosome, nucleus, nucleolus, cytoplasm, and membrane. The advent of these predictors has provided more precise and efficient tools for mRNA localization research, while also presenting new opportunities and challenges for related fields. There is still significant room for improvement in the predictive performance of these computational methods, especially for multi-label prediction. There are currently four predictors, namely DM3Loc developed by Wang et al. [39], Clarion developed by Bi et al. [40], MRSLpred developed by Choudhury et al. [41], and Wang’s method [42], which address the reality that mRNA may exist at multiple subcellular localizations.

The mRNA sequence harbors intricate structures and intermolecular interactions. Considering the potential limitations of existing predictors, notably the inability to accurately predict mRNA presence at multiple subcellular localizations, traditional feature extraction methods also fail to adequately capture and represent sequence information. This study proposes a novel multi-label predictor, DRpred, for mRNA subcellular localization prediction. Initially, Bayesian networks [43] are employed to learn the relationships between labels, thereby capturing the dependencies among them. Subsequently, the Word2vec [44] method is utilized for mRNA sequence feature extraction, incorporating the label dependencies as model input. Finally, a neural network combining BiLSTM and an attention mechanism is constructed to capture the features of various mRNA sequences for predicting mRNA subcellular localization. The 10-fold cross-validation results on the training-validation set demonstrate that DRpred achieves a competitive predictive performance. Furthermore, independent testing results indicate that DRpred outperforms the current state-of-the-art methods in predicting accuracy across various subcellular compartments. This suggests that DRpred holds broad potential for application in future bioinformatics research.

## 2. Materials and Methods

### 2.1. Datasets

Constructing an accurate and reliable dataset is a crucial step in developing an effective predictor. To ensure that subsequent comparison results are impartial, this study utilizes a dataset that is entirely consistent with the current state-of-the-art mRNA localization predictor, Clarion. The dataset was constructed by Bi et al. [40] in 2022, where all data (including mRNA sequences and subcellular localization annotation information) were sourced from the RNALocate database (version 2.0), which was last updated in June 2021. Initially, Bi et al. conducted a statistical analysis on 84,792 mRNA sequences retrieved from the RNALocate database, each containing subcellular localization annotations. Entries with incomplete annotations and those with fewer than 3000 occurrences were removed. Subsequently, based on the multiple localization of mRNA sequences in the transcriptome, the mapping relationship between mRNA and different subcellular localizations was redefined, thereby constructing a multi-label mRNA subcellular localization dataset. Furthermore, to mitigate the impact of redundant sequences on the predictor performance, the dataset employed CD-HIT-EST [45] to remove sequences with 80% homology, ensuring that the similarity between any two nucleotide sequences is less than 80%. Ultimately, a total of 36,971 mRNA sequences were obtained, annotated with nine subcellular localizations, including the exosome, nucleus, cytosol, chromatin, nucleoplasm, ribosome, nucleolus, cytoplasm, and membrane.

#### 2.1.1. mRNA Sequences Distribution and Nucleotide Composition Analysis

**Figure 1:**
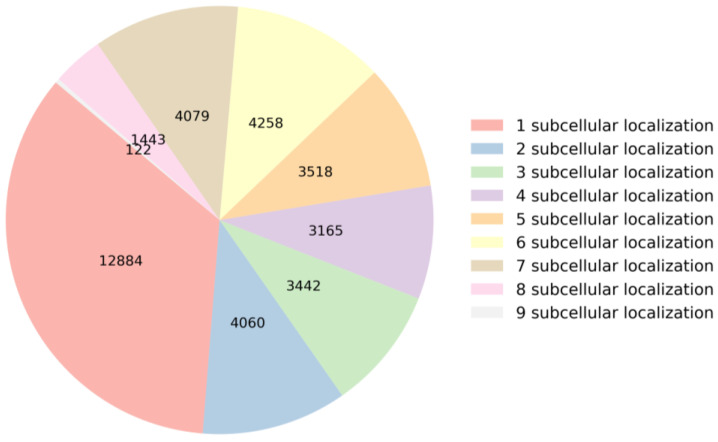
Statistics on the number of labels in the dataset.

When an mRNA sequence is present in a specific subcellular location, it may play a significant role in the cellular function of that subcellular location. If an mRNA sequence is found in multiple subcellular locations, it indicates that the mRNA may be involved in multiple cellular functions or biological processes. To gain a deeper understanding of the relationship between mRNA sequences and subcellular localization, this section analyzes the distribution of mRNA sequences across subcellular locations and the nucleotide composition within the sequences in the dataset. This analysis serves as the first and crucial step in building a predictor. Specifically, the dataset comprises a total of 36,971 mRNA sequences. They are categorized based on the number of subcellular locations they are annotated with, as illustrated in Figure 1. Among these sequences, 12,884 mRNA sequences have only one label, 4060 mRNA sequences have two labels, 3442 mRNA sequences have three labels, 3165 mRNA sequences have four labels, 3518 mRNA sequences have five labels, 4258 mRNA sequences have six labels, 4079 mRNA sequences have seven labels, 1443 mRNA sequences have eight labels, and 122 mRNA sequences have nine labels. These distributions of subcellular locations provide crucial foundational data for constructing a predictor.

Based on the subcellular localization distribution of the sequences, the dataset contains 31,448 mRNA sequences localized in the exosome, 21,439 mRNA sequences localized in the nucleus, 14,237 mRNA sequences localized in the nucleoplasm, 14,328 mRNA sequences localized in the chromatin, 4016 mRNA sequences localized in the cytoplasm, 11,124 mRNA sequences localized in the nucleolus, 16,312 mRNA sequences localized in the cytosol, 6739 mRNA sequences localized in the membrane, and 8680 mRNA sequences localized in the ribosome. This study divides the dataset into a training-validation set and an independent test set in a 9:1 ratio. The specific distribution of these sequences across the nine subcellular locations is presented in Table 1.

**Figure 2:**
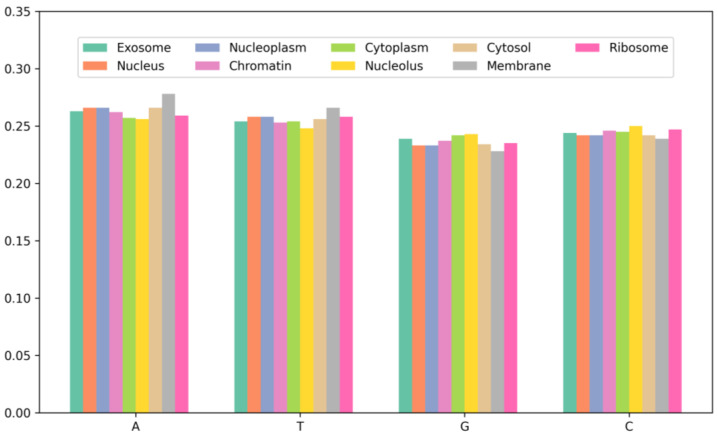
The nucleotide composition of the sequences at each localization in the dataset.

To further investigate the characteristics of mRNA sequences in different subcellular locations, this study conducted a statistical analysis of the single-nucleotide composition of mRNA sequences across nine subcellular locations, as shown in Figure 2. By comparing the distribution of nucleotide content across the nine subcellular locations, it can be observed that the environment and physiological state of the different subcellular locations may influence the composition and expression of mRNA. For instance, certain RNA-binding proteins may bind and regulate mRNA transcription and splicing within the nucleus, thereby affecting mRNA composition and expression. Additionally, the similarity in nucleotide distribution among the cytoplasm, ribosome, and nucleolus is due to the fact that the cytoplasm and ribosome are key locations for protein synthesis, where mRNA needs to be translated into proteins. Furthermore, studies on mRNA have indicated that they are enriched in the nucleus, cytoplasm, and ribosome compared to other subcellular locations, which aligns with the results of the nucleotide content analysis in this study. The experimental results suggest that predictive models for mRNA subcellular localization need to consider the biological characteristics of different subcellular locations in order to more accurately predict the localization of mRNA sequences.

According to the results in Figure 2, mRNA exhibits differences in nucleotide content distribution across various subcellular compartments. For instance, mRNA in the membrane has the highest A and T content at 27.8% and 26.6%, respectively, while the lowest C and G content at 22.8% and 23.9%, respectively. This discrepancy may be related to the biological functions and metabolic demands of mRNA within the membrane. Additionally, mRNA in the nucleolus has the highest G and C content, reaching 49.3%, which exceeds other subcellular locations. Studies have indicated that increasing the G and C content can enhance mRNA stability and translation efficiency. Therefore, it can be inferred that there is a correlation between nucleotide content distribution in the subcellular compartments and mRNA sequence distribution. This analysis aids in gaining a deeper understanding of the mRNA sequence characteristics across different subcellular locations, providing a foundation and guidance for further research.

#### 2.1.2. Label Correlation Analysis

**Figure 3:**
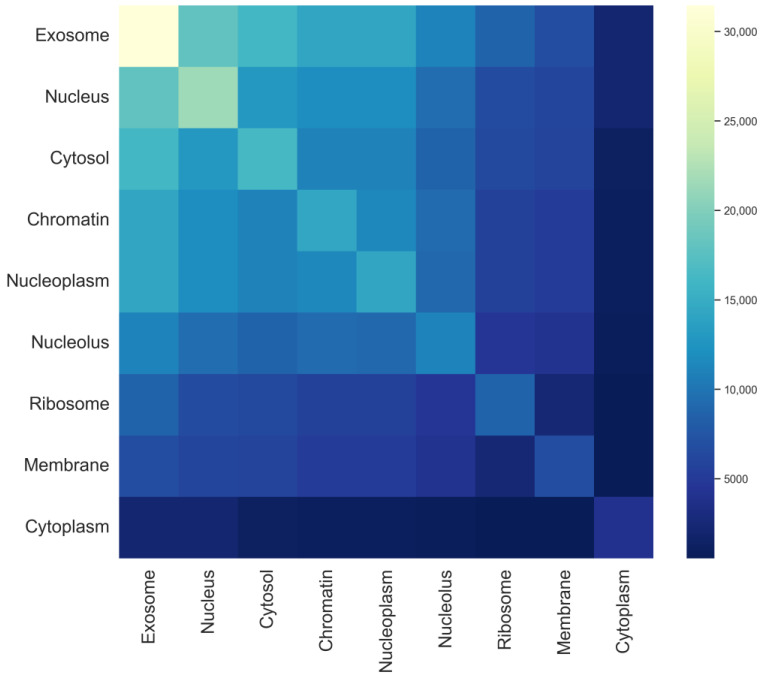
Tag set heat map for each subcellular localization in the mRNA sequence.

In multi-label prediction tasks, label dependencies and semantic representations are critical issues. In the dataset, reliable labels can provide valuable information for predicting other labels. If the predictor can effectively utilize this information, the predictive performance will significantly improve. In this study, a heatmap of the mRNA sequence label set is plotted, as shown in Figure 3. The darkness of the colors reflects the frequency of occurrence of different labels in the dataset. Lighter colors indicate higher frequencies of occurrence of the label in the dataset, whereas darker colors indicate lower frequencies. By observing the heatmap, it can be noted that the exosome, nucleus, and cytosol have relatively higher occurrence frequencies in the dataset, and their color variation trends are similar, suggesting potential correlations. For example, most mRNA sequences present in the exosome also appear in the nucleus and cytosol, whereas mRNA sequences found in the cytoplasm rarely coexist in both the nucleus and cytosol. In contrast, the ribosome, membrane, and cytoplasm have relatively lower frequencies, indicating their presence in only a few samples. The model will formulate corresponding processing strategies based on these distribution patterns, such as adjusting model parameters or employing different label weights, to enhance the learning effectiveness of the multi-label model.

Based on the data distribution, Bayesian networks are utilized to obtain the marginal probabilities and co-occurrence probabilities between the subcellular localization labels, which serve as prior knowledge. The marginal probability indicates the probability of each label appearing independently, as shown in Table 2. For instance, the marginal probability of the exosome is the highest at 0.188, indicating its highest frequency of occurrence in the dataset. The marginal probabilities of the nucleoplasm and chromatin are very close, at 0.128 and 0.129, respectively, suggesting a similarity in their occurrence probabilities under the same conditions, indicating a potential correlation between them. Additionally, the marginal probability of the cytoplasm is 0.020, indicating a low probability of mRNA sequences appearing in the cytoplasm. However, since marginal probabilities cannot directly reflect the dependencies and nonlinear relationships between labels, detailed relationships between the labels cannot be accurately inferred based solely on marginal probabilities. Therefore, more data and analysis are needed to accurately understand the relationships between the labels.

**Figure 4:**
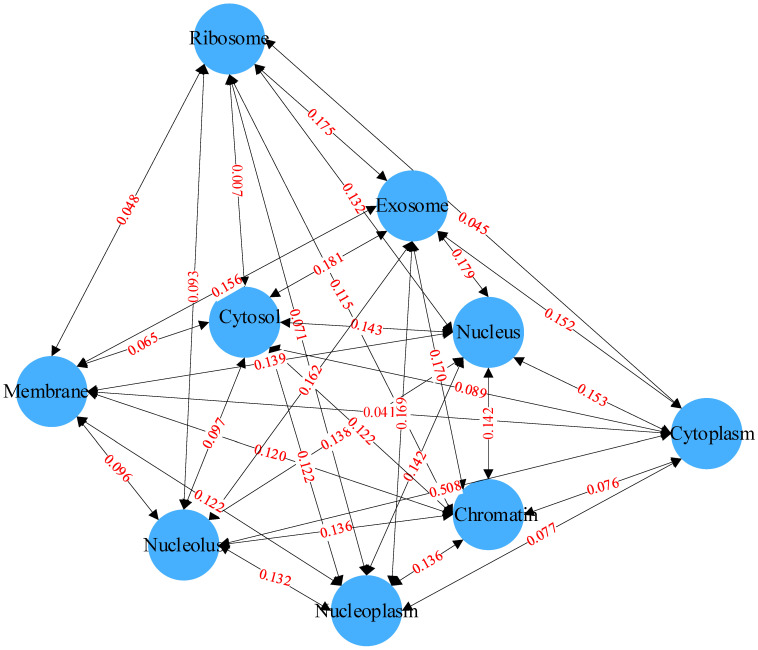
Relationships between labels based on Bayesian networks.

Co-occurrence statistics and distribution representations are jointly used to describe associations in the label set. The co-occurrence statistics reflect the probability of co-occurrence of two or more subcellular locations, and the distribution representation describes the co-occurrence of two or more subcellular locations in the entire label set. By computing the co-occurrence probability, the probability of a certain label appearing given the presence of other labels can be determined. This probability can be used to describe the correlation between labels. As shown in Figure 4, for example, it is evident that the co-occurrence probability of exosome and cytosol is 0.181, reaching the highest co-occurrence probability in the label set. This implies that, knowing an mRNA is distributed in the exosome, there is approximately an 18.1% chance that it is also distributed in the cytosol. Compared to the co-occurrence probabilities between the exosome and the other eight labels, which are all above 0.152, the co-occurrence probability of cytoplasm with the other labels is very low due to the scarcity of cytoplasm labels, with a likelihood of co-occurrence with the other labels of approximately 1%. These rich dependencies between labels will serve as important information for downstream prediction modules, aiding the model in more accurately predicting the distribution of subcellular localization.

### 2.2. Network Framework of DRpred

**Figure 5:**
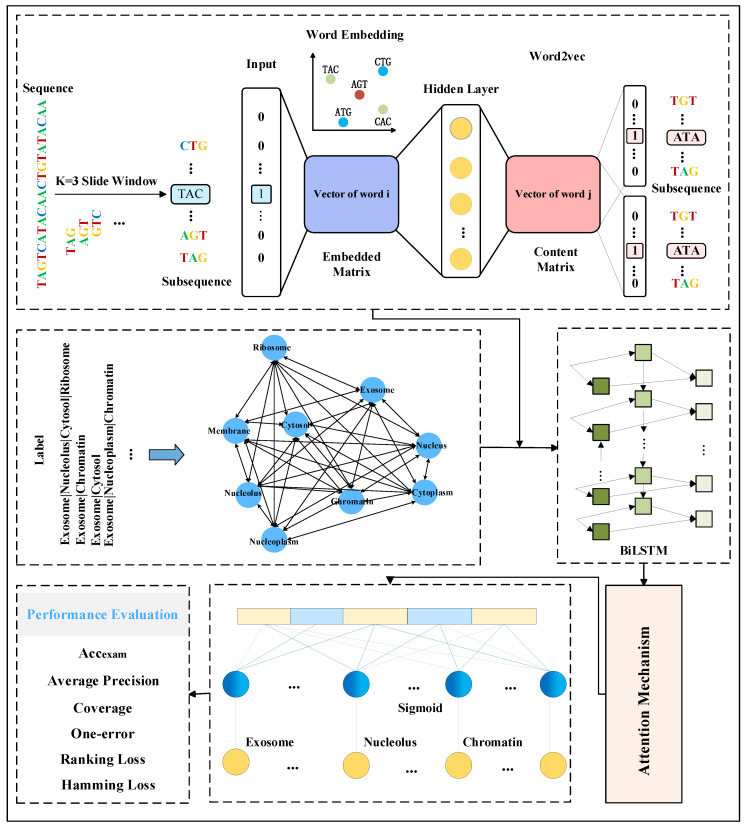
The overall architecture and prediction workflow diagram of the DRpred predictor. The predictor initially employs a sliding window approach to partition mRNA sequences into subsequences, which are then inputted into the Word2vec model for embedding encoding. Simultaneously, a Bayesian network is constructed using the existing mRNA subcellular localization label set as prior information for the mRNA sequences. Specifically, during the training process, the co-occurrence probabilities of each mRNA sequence’s corresponding labels are extracted from the Bayesian network. Subsequently, the probabilities of irrelevant labels are averaged, and a multi-label mean vector is used as the prior information. Finally, the prior information is fused with the mRNA sequence features extracted by Word2vec, and a multi-label subcellular predictor is constructed using a BiLSTM network combined with an attention mechanism.

In order to explore the mRNA sequence features and fully exploit the dependencies among its labels, this study proposes a multi-label mRNA subcellular localization predictor, DRpred, based on BiLSTM combined with an attention mechanism. The overall architecture and basic workflow of the predictor are illustrated in Figure 5.

### 2.3. Feature Extraction

**Figure 6:**
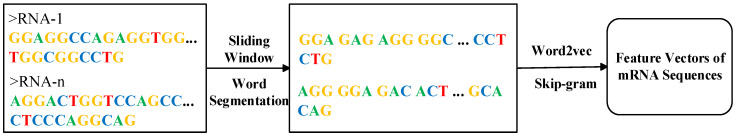
Feature encoding workflow diagram of mRNA sequences in DRpred.

Encoding the original mRNA sequence data to obtain discriminative features is the primary challenge in constructing subcellular localization predictors based on deep learning. To address this issue, for a given mRNA sequence, the DRpred first segments it into multiple subsequences using a sliding window approach. When a sequence of length L is segmented with a sliding window of size k and a step size of 1, L−k+1 subsequences are obtained. In practical applications, if the value of k is too small, it will result in a very large number of subsequences after segmentation, making the analysis process complex and difficult. Conversely, if the value of k is too large, it may lead to excessive information loss. Therefore, based on experimental comparison results, DRpred employs a sliding window with a step size of 1 and a window size of k = 3 (i.e., using 3 nucleotides) for segmentation. Subsequently, the Word2vec model is trained using all the mRNA sequences in the dataset, and the Skip-gram method within this model is employed to learn the co-occurrence statistics and distribution representations of the mRNA subsequences. The Word2vec model treats each subsequence as a word and each sequence as a sentence for training. The entire feature encoding process of DRpred is illustrated in Figure 6. First, mRNA sequences from the entire dataset are used as a corpus and segmented into subsequences of equal length using a sliding window. These subsequences are then trained using the Word2vec model to learn their distribution representations. Finally, the resulting features are used as input for the deep learning prediction model. This encoding scheme effectively leverages the hidden advanced features within the sequence information.

The key method is Skip-gram. Skip-gram is trained by predicting the surrounding subsequences of a central mRNA subsequence. After training, the weight matrix of the hidden layer is obtained, which represents the word vectors. For a given sequence $\left(w_{1},w_{2},\ldots ,w_{l-k+1}\right)$, the objective of the model during training is to adjust the values of the word vectors to maximize the average logarithmic probability (i.e., maximize prediction accuracy).

(1) $$f=max\frac{1}{N}\sum_{n=1}^{N}\sum_{-c\leq m\leq c}\log P\left(w_{n+m}|w_{n}\right)$$

where c represents the distance to the central subsequence, whose logarithmic probability distribution can be defined as follows:

(2) $$\log P\left(w_{0}|w_{i}\right)=\log\frac{e^{v_{w_{0}}^{’T}v_{w_{i}}}}{\sum_{w=1}^{W}e^{v_{w}^{’T}v_{w_{i}}}}$$

where $v_{w}$ and $v_{w}^{’}$ are the input and output vectors of the word (i.e., subsequence) w, respectively. T denotes the transpose operation and W is the size of the training dictionary when training the mRNA sequence.

### 2.4. Extraction of Label Relationships

Despite the promising performance of deep learning in single-label subcellular localization tasks, maintaining high predictive accuracy in complex multi-label tasks remains a challenging issue. To address this issue, this study employs Bayesian networks to directly learn the interrelationships between the different subcellular localization labels. As a probabilistic graphical model, Bayesian networks can effectively capture the correlations between variables in the data and will be utilized in this study to extract dependencies (co-occurrences) between the labels. When constructing a Bayesian network, it is first necessary to determine the nodes (i.e., variables) and establish the set of nodes $X=X_{1},X_{2},\ldots ,X_{n}.$ In this study, the nodes represent a specific subcellular location of mRNA sequence distribution, where each node corresponds to a site label. Subsequently, it is necessary to ascertain the conditional dependency relationships between the labels, which are the edges connecting the nodes. These edge relationships represent the probabilistic connections between labels. For instance, if label $X_{i}$ and label $X_{j}$ co-occur, an edge is established from $X_{i}$ to $X_{j}$, indicating a dependency relationship between $X_{i}$ and $X_{j}$. Subsequently, a co-occurrence probability table is determined for each site label $X_{i}$. This table lists the co-occurrence probabilities between all possible values of a given site label and the possible values of other site labels. Finally, the Bayesian network is trained to learn the co-occurrence probabilities for each node and to infer the probabilistic relationships between the variables through the assumption of conditional independence. During subsequent training, the co-occurrence probabilities of each mRNA sequence’s corresponding labels are extracted from the Bayesian network. The probability values for these labels are retained, and the average of the remaining unrelated label probabilities is calculated to serve as prior information. For instance, if an mRNA sequence is labeled with exosome and cytoplasm, the probabilities for the exosome and cytoplasm are retained from the co-occurrence probability distribution, and the mean of the probabilities for the other seven subcellular localization labels are computed. This resulting multi-label mean vector will be used as prior knowledge. The multi-label mean vector is concatenated with the sequence features extracted by Word2vec. The resulting features are then used as inputs for the downstream prediction models. Furthermore, since the mRNA independent test set used in independent testing lacks any information related to its subcellular localization distribution, the independent test set sequences are initially represented by feature vectors extracted using Word2vec. Subsequently, these vectors are compared with the feature vectors in the training-validation set using nearest neighbor computation to obtain the sequence features in the training-validation set that are most similar to those of the independent test set sample. Then, prior information on the labels of the most similar sequences in the training-validation set is utilized as prior knowledge for the sample, addressing the issue of independent test samples lacking prior label information.

### 2.5. Multi-Label Prediction

Although manually extracted features possess interpretability, their inclusion of significant redundant information may lead to training biases, consequently impacting the predictive performance of the predictor. Furthermore, while deep learning-based models can automatically encode sequences, perform feature extraction, and classification, they lack the interpretability in learning crucial features. In order to construct an efficient mRNA subcellular localization predictor, the DRpred integrates two deep learning methods—BiLSTM and attention mechanism. Initially, BiLSTM is employed to capture long-term information from the input sequence feature vectors. Subsequently, the self-attention mechanism is utilized to extract crucial positional information from the sequence features. This approach automatically extracts useful features from the sequence while simultaneously handling both forward and backward information within the sequence.

Specifically, each mRNA sequence’s feature vector obtained through the Word2vec model is combined with the model’s prior knowledge (dependencies between labels) and fed into the BiLSTM model. Subsequently, the BiLSTM model sequentially processes each time step. At each time step, the model takes the current input vector and the hidden state from the previous time step as input, computes the current time step’s hidden state, and progresses. Then, the attention mechanism automatically focuses on the mRNA sequence feature components relevant to subcellular localization. It calculates a weighted average of all positions in the input sequence to obtain the important feature vectors corresponding to subcellular localization. Finally, more representative feature representations extracted by the attention mechanism are utilized as inputs for fully connected layers for classification prediction, yielding the predicted subcellular localization.

### 2.6. Bidirectional Long Short-Term Memory

**Figure 7:**
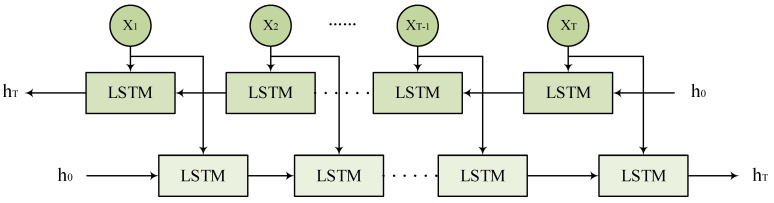
BiLSTM network architecture.

To more effectively capture the dependency relationships of features in the input predictors, this study introduces the Bidirectional Long Short-Term Memory network (BiLSTM) as a key component. As shown in Figure 7, BiLSTM captures bidirectional contextual dependencies by applying two independent LSTM layers to the forward and backward information flow of the input features. For a given set of input features $X=\left[x_{1},x_{2},x_{3},\ldots ,x_{T}\right]$, the forward LSTM generates the forward hidden states $\underset{h_{t}}{\to }$ from time step 1 to T, while the backward LSTM generates the backward hidden states $\underset{h_{t}}{\leftarrow }$ from time step T to 1. Finally, BiLSTM concatenates the forward and backward hidden states at each time step t for prediction tasks, further improving the performance of mRNA subcellular localization.

### 2.7. Performance Evaluation Metrics

Due to the necessity of considering the predictive performance across all the labels, the evaluation metrics for multi-label prediction tasks are more complex than those for single-label tasks. In this study, six commonly used evaluation metrics in the RNA subcellular localization field are selected to assess the predictive performance of DRpred. These six evaluation metrics include example-based accuracy (Acc_exam_), Average Precision, Coverage, One-error, Ranking Loss, and Hamming Loss. Their computation formulas are as follows:

(3) $${Acc}_{exam}=\frac{1}{r}\sum_{i=1}^{r}\frac{P_{i}\cap T_{i}}{P_{i}\cup T_{i}}$$

(4) $$\mathrm{Average}\mathrm{Precision}=\frac{1}{r}\sum_{i=1}^{r}\frac{1}{\left|T_{i}\right|}\times \sum_{t\in T_{i}}\frac{\left|\{t’Rank_{c}{(s}_{i},t^{\prime })\leq Rank_{c}{(s}_{i},t),t^{\prime }\in T_{i}\}\right|}{{\mathrm{Rank}}_{c}{(s}_{i},t)}$$

(5) $$Coverage=\frac{1}{r}\sum_{i=1}^{r}{max}_{t^{\prime }\in T_{i}}{Rank}_{c}\left(s_{i},t^{\prime }\right)-1$$

(6) $$One-error=\frac{1}{r}\sum_{i=1}^{r}I\left({argmax}_{t^{\prime }\in T_{i}}c\left(s_{i},t^{\prime }\right)\notin Y_{i}\right)$$

(7) $$Ranking\;Loss=\frac{1}{r}\sum_{i=1}^{r}\frac{1}{\left|T_{i}\right|\left|{T_{i}}^{\prime }\right|}I\left(c\left(s_{i},t^{\prime }\right)\leq c\left(s_{i},t^{\prime \prime }\right),t^{\prime }\in T_{i},t^{\prime \prime }\in {T_{i}}^{\prime }\right)$$

(8) $$Hamming\;Loss=\frac{1}{r}\sum_{i=1}^{r}\frac{1}{q}\left|P_{i}∆Y_{i}\right|$$

where $c\left(\cdot \right)\;$represents a multi-label classifier, $\left(s_{i},T_{i}\right)\left\{1\leq i\leq r\right\}\;$represents a multi-label mRNA sequence prediction instance, where $T_{i}$ and $P_{i}$ represent the true label set and the predicted label set for the mRNA sequence, respectively. ${T_{i}}^{\prime }$ is the complement set of $T_{i}$. ${Rank}_{c}\left(s,t\right)$ denotes the descending rank of t in $T_{i}$. When q is the cardinality of $T_{i}$, |·| represents the cardinality of the set, ∆ denotes the symmetric difference between two sets, and $I\left(\cdot \right)\;$is used to calculate the number of satisfying conditions. Acc_exam_ is calculated by comparing the ratio of the intersection to the union of the predicted and true label sets for each instance, reflecting the overall predictive accuracy of the classifier across all test instances. Average Precision is calculated by averaging the precision values at different recall levels across all instances. Specifically, for each instance, the Average Precision takes into account the ranks of the relevant labels and assesses how well the classifier places these labels at the top of the ranking. Coverage measures how well a classifier explores the label space by averaging the rank of the highest-ranked true label across all instances, with lower values indicating better exploration. One-error measures the proportion of instances where the highest-ranked predicted label is not among the true labels, with a lower value indicating better classification performance. Ranking Loss quantifies the average number of label pairs where a true label is ranked lower than a false label, with lower values indicating better ranking performance. Hamming Loss measures the average fraction of incorrect labels by comparing the predicted label set to the true label set for each instance, with lower values indicating better performance. Considering these metrics collectively allows for a more comprehensive evaluation of a predictor’s performance and provides guidance for improving the predictor’s performance.

## 3. Results and Discussion

### 3.1. Comparison of Different Sequence Coding Schemes

In the field of bioinformatics, particularly in sequence-based explorations, deep learning models cannot directly recognize character-based sequence data. Therefore, it is essential to encode the sequences initially, converting them into numerical vectors before inputting them into prediction models. Different sequence encoding schemes reflect various features of the sequences, which have different impacts on mRNA subcellular localization prediction. Hence, selecting an appropriate sequence encoding scheme is a crucial step.

To improve the accuracy of mRNA subcellular localization, this study explored different sequence encoding schemes. Through 10-fold cross-validation, the traditional K-mer encoding method was compared with Word2vec models with different sliding window sizes, comprehensively evaluating the impact of different feature encoding schemes on model prediction performance. The experimental results are presented in Table 3, where it is observed that using the traditional encoding methods, K-mer and Word2vec, with a sliding window size of 1 results in poor performance during model training, with Acc_exam_ values of 0.649 and 0.654, respectively. However, when the window size is set to 3, the Word2vec model obtains the best performance during training, with an Acc_exam_ value 0.018 higher than that of the K-mer method and 0.013 higher than that of the sliding window size of 2. The experimental results demonstrate that the Word2vec model with a window size of 3 can better represent mRNA sequence information.

Specifically, the feature representation generated using the K-mer method is associated with specific elements in the mRNA sequence, but it fails to consider the global semantic relationships within the entire sequence thus neglecting the original order information of the mRNA sequence. Additionally, the K-mer method tends to lose information when dealing with longer sequences or sequences with low frequencies. In contrast, Word2vec encoding learns the semantic relationships between the mRNA subsequences and maps these subsequences to an abstract vector space, utilizing low-dimensional vectors to represent each subsequence. This encoding method is more suitable for various sequence data of different lengths, thereby enhancing the model’s generalization capability. Hence, this study opts for Word2vec encoding with a window size of 3 to obtain more enriched and semantically meaningful feature vectors, resulting in a more concise representation of the sequences.

### 3.2. Comparison of Different Network Architectures

Different deep neural network architectures have a significant impact on the predictive performance and generalization ability of predictors. In this study, various network architectures were compared, analyzed, and selected through 10-fold cross-validation. First, LSTM was employed as the baseline model, and fine-tuning was conducted on this network. Subsequently, attention mechanisms were integrated into the LSTM model for retraining and fine-tuning. Finally, an attempt was made to combine BiLSTM with the attention mechanisms. The experimental results demonstrate that, as shown in Table 4, the predictive model utilizing BiLSTM combined with attention mechanisms exhibits outstanding performance in the mRNA subcellular localization prediction tasks. In comparison with the other two network architectures, this model achieves the highest Acc_exam_ and Average Precision, reaching 0.703 and 0.753, respectively, while yielding the lowest Ranking Loss and Hamming Loss, with values of 0.239 and 0.154, respectively. Compared to LSTM, the model combining BiLSTM with attention mechanisms improves Acc_exam_ and Average Precision by 0.032 and 0.026, respectively. Furthermore, compared to the LSTM model combined with attention mechanisms, this model improves Acc_exam_ and Average Precision by 0.019 and 0.014, respectively. These results indicate that, compared to the LSTM model, which can only read input sequences in one direction, BiLSTM has the capability to read input sequences in both forward and backward directions simultaneously, enabling better capture of the sequence context information. Additionally, the attention mechanism can focus on important parts of the sequence features related to the interactions between different subcellular locations, thereby allocating different weights. This allows the model to better learn the dependencies between the training samples and the labels, enhancing the accuracy of subcellular location prediction.

### 3.3. Performance Comparison with Existing State-of-the-Art Predictors

In order to provide a more objective assessment of the predictor’s performance, this study compares the DRpred predictor proposed here with several existing mRNA subcellular localization predictors. Currently, there are the following four mRNA subcellular localization predictors involved in multi-label prediction: the method proposed by Wang et al. [42], DM3Loc, Clarion, and MRSLpred. Firstly, DRpred is compared with these four multi-label predictors. Since DRpred and Clarion use exactly the same dataset, the prediction results for all nine subcellular locations are compared on an independent test set. Additionally, in the multi-label prediction of DM3Loc and MRSLpred models, only the five subcellular locations (cytosol, exosome, nucleus, ribosome, and membrane) overlap with the predicted locations of DRpred, whereas in the multi-label predictor proposed by Wang et al., the overlapping subcellular locations with DRpred are cytosol, exosome, nucleus, ribosome, and cytoplasm. Given the difference in the five subcellular locations covered by the DM3Loc, MRSLpred, and Wang et al.’s predictors in the experiments, DRpred is separately compared with these predictors.

As shown in the experimental results in Table 5 and Table 6, DRpred, Clarion, MRSLpres, and DM3Loc achieved Acc_exam_ values of 0.731, 0.722, 0.642, and 0.441, respectively, when predicting the subcellular locations of cytosol, exosome, nucleus, ribosome, and membrane on an independent dataset. Comparatively, DRpred achieved the highest Acc_exam_ value, indicating that it predicted the most correct labels for this subcellular location prediction task. Additionally, DRpred also performed well in terms of Average Precision, reaching 0.771. In comparison with Clarion and the method proposed by Wang et al., DRpred achieved an Acc_exam_ value of 0.752 on the independent dataset for the prediction of cytosol, exosome, nucleus, ribosome, and cytoplasm subcellular locations, significantly outperforming the other two methods. It is noteworthy that the performance of DRpred in terms of Average Precision and One-error, which are used to evaluate the quality of classifier ranking, was comparable to the Clarion method and did not achieve significantly better results. This may be attributed to issues such as sample imbalance and mutual interference between labels in the dataset. Overall, these results suggest that the predictive performance of DRpred in multi-label tasks is generally superior to several of the current state-of-the-art mRNA subcellular localization predictors.

Currently, most existing mRNA subcellular localization predictors only support single-label prediction. Therefore, in this section, the unit-point prediction performance of DRpred was compared with other advanced methods for mRNA localization prediction. The methods accessible through web servers and open-access standalone code in GitHub were utilized for prediction and comparison, including iLoc-mRNA, mRNALoc, mRNALocator, and MSLP, to facilitate reliable performance comparison. Specifically, the mRNA sequences from the independent test set were uploaded to the network servers of these methods or open-access standalone code in GitHub, and then the predicted labels were compared with the true labels. The results in Table 7 demonstrate that DRpred outperforms other methods in predicting subcellular locations such as cytoplasm, cytosol, exosome, and ribosome, and nearly maintains an accuracy of over 80% in all nine subcellular locations. Furthermore, in the prediction of exosome, cytoplasm, and membrane by DRpred, the accuracies reached 94.00%, 93.02%, and 90.58%, respectively. These comparative results further confirm the outstanding predictive ability of DRpred in single-site tasks.

## 4. Conclusions

The study of subcellular mRNA localization distribution provides important theoretical foundations for understanding mRNA function and the regulation of related diseases. However, traditional handcrafted feature extraction methods cannot fully capture and express the hidden information in mRNA sequences due to their complex structures and interactions. Moreover, a majority of the mRNA sequences have multiple subcellular location labels, and the complexity of the label space often makes it difficult for models to fully learn them, resulting in reduced predictive capability. To fully exploit the hidden feature information of mRNA sequences and utilize the dependencies between labels, this study proposes a novel multi-label subcellular mRNA predictor called DRpred. This predictor can simultaneously predict the distribution of nine subcellular locations, including exosome, nucleus, nucleoplasm, chromatin, cytoplasm, nucleolus, cytosol, membrane, and ribosome, within mRNA sequences. Specifically, DRpred first segments mRNA sequences using a sliding window and encodes them using a Word2vec model for feature extraction. Next, it models the label set using Bayesian networks to capture the relationships between the different labels. By retaining the probabilities of the corresponding labels for each sequence and averaging the probabilities of other irrelevant labels, these probabilities serve as the prior knowledge of the model. Then, these prior knowledge values are concatenated with the feature vectors and input into a BiLSTM model. Subsequently, an attention mechanism is applied to calculate the weights of the model’s output, which are then fed into a fully connected layer for subcellular location classification prediction. DRpred maps the mRNA sequence feature vectors and label relationships to a relevant space, effectively utilizing label dependencies and fully exploring the hidden feature information within the mRNA sequences, thereby enhancing the model’s predictive capability. Experimental results demonstrate that DRpred achieves higher accuracy in multi-label prediction within the mRNA subcellular localization. Therefore, DRpred will serve as a useful tool for rapid analysis based on mRNA sequences and predicting their subcellular localization distributions.

## Figures and Tables

**Table 1:** Statistical distribution of mRNA sequences at nine subcellular localizations in the dataset.

Subcellular Localization	Total	Training-Validation Set	Independent Test Set
Exosome	31,448	28,304	3144
Nucleus	21,439	19,301	2138
Nucleoplasm	14,237	12,807	1430
Chromatin	14,328	12,893	1435
Cytoplasm	4016	3597	419
Nucleolus	11,124	10,000	1124
Cytosol	16,312	14,686	1626
Membrane	6739	6047	692
Ribosome	8680	7796	884

**Table 2:** Marginal probabilities for each label of mRNA sequences.

Subcellular Localization	Exosome	Nucleus	Nucleoplasm	Chromatin	Nucleolus	Membrane	Ribosome	Cytosol	Cytoplasm
Marginal Probability	0.188	0.153	0.128	0.129	0.104	0.065	0.075	0.137	0.020

**Table 3:** Performance comparison of different feature encoding schemes on the training-validation set.

	K-mer	Word2vec (k = 1)	Word2vec (k = 2)	Word2vec (k = 3)
Acc_exam_	0.649	0.654	0.662	0.667
Average Precision	0.711	0.717	0.722	0.725
Coverage	3.900	3.840	3.771	3.730
One-error	0.556	0.550	0.544	0.536
Ranking Loss	0.318	0.311	0.304	0.299
Hamming Loss	0.175	0.173	0.170	0.169

**Table 4:** Performance comparison of different network architectures on the training-validation set.

	LSTM	LSTM + Attention	BiLSTM + Attention
Acc_exam_	0.671	0.684	0.703
Average Precision	0.727	0.739	0.753
Coverage	3.690	3.624	3.477
One-error	0.531	0.521	0.509
Ranking Loss	0.293	0.261	0.239
Hamming Loss	0.167	0.162	0.154

**Table 5:** Comparison of multi-label prediction performance with DM3Loc, MRSLpred, and Clarion predictors at five subcellular localizations on the independent test set (membrane, cytosol, exosome, nucleus, ribosome).

	DM3Loc	MRSLpred	Clarion	DRpred
Acc_exam_	0.441	0.642	0.722	0.731
Average Precision	0.618	0.712	0.769	0.771
Coverage	3.957	3.736	3.019	2.998
One-error	0.869	0.654	0.463	0.451
Ranking Loss	0.533	0.423	0.204	0.182
Hamming Loss	0.330	0.268	0.150	0.098

**Table 6:** Comparison of multi-label prediction performance on five subcellular localizations (cytoplasm, cytosol, exosome, nucleus, ribosome) with predictors proposed by Wang et al. [42]. and Clarion on the independent test set.

	Wang’s	Clarion	DRpred
Acc_exam_	0.281	0.745	0.752
Average Precision	0.679	0.767	0.767
Coverage	4.516	3.127	3.069
One-error	0.882	0.445	0.447
Ranking Loss	0.716	0.241	0.194
Hamming Loss	0.375	0.146	0.097

**Table 7:** Comparison of prediction performance with other predictors at single subcellular localizations on the independent test set.

	iLoc-mRNA	mRNALoc	mRNALocator	MSLP	DRpred
Chromatin	-	-	-	-	82.14%
Cytoplasm	-	54.88%	38.90%	84.05%	93.02%
Cytosol	-	-	-	78.50%	80.37%
Exosome	-	-	-	80.00%	94.00%
Membrane	-	-	-	-	90.58%
Nucleolus	-	-	-	-	84.53%
Nucleoplasm	-	-	-	-	82.01%
Nucleus	-	55.18%	57.42%	85.40%	79.71%
Ribosome	73.41%	-	-	79.30%	85.67%

“-”: non-applicable.

## Data Availability

The source codes and data for DRpred are available at https://github.com/YangL-Coder/DRpred (accessed on 21 August 2024).
